# Deciphering the roles of unknown/uncharacterized genes in plant development and stress responses

**DOI:** 10.3389/fpls.2023.1276559

**Published:** 2023-11-23

**Authors:** Xi Wang, Baoshan Wang, Fang Yuan

**Affiliations:** Shandong Provincial Key Laboratory of Plant Stress, College of Life Sciences, Shandong Normal University, Ji’nan, Shandong, China

**Keywords:** function, growth, stress resistance, structure, unknown/uncharacterized genes

## Abstract

In recent years, numerous genes that encode proteins with specific domains that participate in different biological processes or have different molecular functions have been identified. A class of genes with typical domains whose function has rarely been identified and another type of genes with no typical domains have attracted increasing attentions. As many of these so-called as unknown/uncharacterized (U/U) genes are involved in important processes, such as plant growth and plant stress resistance, there is much interest in deciphering their molecular roles. Here, we summarize our current understanding of these genes, including their structures, classifications, and roles in plant growth and stress resistance, summarize progress in the methods used to decipher the roles of these genes, and provide new research perspectives. Unveiling the molecular functions of unknown/uncharacterized genes may suggest strategies to fine-tune important physiological processes in plants, which will enrich the functional network system of plants and provide more possibilities for adaptive improvement of plants.

## Introduction

The development of modern molecular biology tools has accelerated the discovery of genes involved in various biological processes. Many genes have known functions in regulating various physiological processes and mechanisms in plants, such as vegetative growth that the overexpressing of *Lb1G04899* from *Limonium bicolor* improved the salt tolerance of transgenic Arabidopsis under NaCl environment (Liu et al., 2022a; Wang et al., 2022b); flowering time that *CYLIN - DEPENDENT KINASE G2* (*CDKG2*) gene affected flowering time in Arabidopsis (Ma and Chen, 2007; Ma et al., 2015; Zhou et al., 2019); changes in phytohormone status that the NHL family genes of wild soybeans can regulate ABA and MeJA, laying the foundation for potential roles in signal transduction mechanisms (Xu et al., 2020; Zhang et al., 2022b; Zhao et al., 2023a); anther and pollen development that *BcMF19* inhibited pollen development in Chinese cabbage-pak-choi (Huang et al., 2011) and resistance to harsh environments including drought that *TaDTG6-BDel574* regulates the transcription of *TaPIF1* to enhance drought resistance in wheat (Mei et al., 2022); salt that *CycC1* controlled salt tolerance in Arabidopsis by regulating transcriptional regulation of SOS1 (Lu et al., 2023; Ma et al., 2023); diseases that adult-plant resistance (APR) genes played roles in inhibiting the occurrence of wheat rust (Dinglasan et al., 2022) and insect pests that three genes (Cry1Ac-Cry2Ab-EPSPS) in cotton have resistance to lepidopteran insect (Siddiqui et al., 2022). Based on conserved structural domains in their encoded proteins, various gene families are known to regulate different physiological processes, including development, reproduction, and environmental adaptation. For example, members of the SWEET family (containing an MtN3/saliva transmembrane domain) promote ion and sugar transport (Guan et al., 2008; Chen et al., 2010; Slewinski, 2011; Fang et al., 2022; Liu et al., 2022c); members of the WRKY family (containing a WRKY domain) participate in plant defense and aging processes (Silke and Somssich, 2001; Miao et al., 2004; Besseau et al., 2012); and members of the MYB family (containing an MYB domain) are widely involved in development and stress responses.

Genes with established functions are annotated based on the domains in their encoded proteins (defined as structural annotation) and their functions are verified by deletion or overexpression analysis (functional annotation). Genome annotation is primarily based on gene structure, that is, the boundaries of exons/introns and CDS (coding sequences)/UTRs (untranslated regions), at protein-coding loci (Zhang et al., 2022). With the advent of high-throughput sequencing technology, numerous genes have been sequenced and found to encode proteins with unknown/uncharacterized domains. The localizations of these proteins are also uncertain based on structural annotation. Thus, these genes are defined as unknown/uncharacterized (U/U) genes. Although the biological functions of proteins encoded by U/U genes are unclear, these genes occupy a large proportion of genes reported to date (Imtiaz, 2022).

Do U/U genes matter? More and more of these genes have been shown to play important roles in plants, such as controlling growth and development (Wang et al., 2020) and stress resistance (Soda et al., 2013; Qi et al., 2023). It is challenging to classify U/U genes. Here, we focus on recent progress in our understanding U/U genes, including their classifications, methods and functions. We also discuss research methods used to further study U/U genes.

## Classification of U/U genes

Genes with typical domains whose function has rarely been identified and genes with no typical domains whose roles are uncertain were named unknown/uncharacterized (U/U) genes. Here, we classify U/U genes into two types based on the presence or absence of conserved domains.

**Figure 1:**
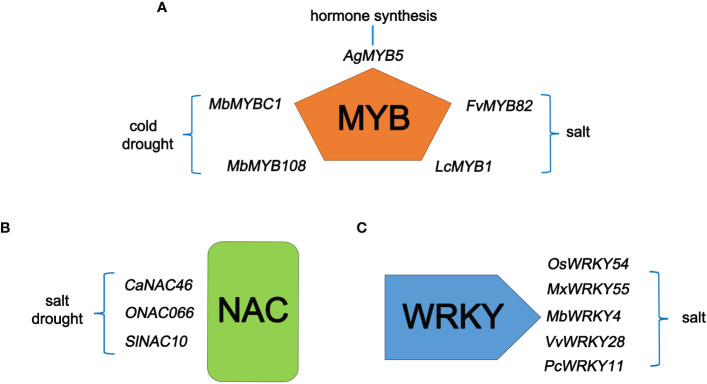
U/U genes with typical conserved domains and their roles in plants. **(A)** Five newly identified MYB type genes and their related functions. **(B)** Two newly discovered NAC family genes and their related functions. **(C)** Three newly isolated WRKY genes and their related functions.

One type is genes with domains but functions have not been identified. Many U/U genes have been identified in food crops and uncultivated plants. Many genes of unknown function contain conserved domains, allowing them to be classified into gene families that encode proteins with known functions. The presence of conserved domains helps researchers predict the roles of U/U genes and provides direction for the functional research of unknown genes. Exogenously overexpressing *MbMYBC1* and *MbMYB108* from *Malus baccata* enhanced the cold and drought resistance of transgenic Arabidopsis (*Arabidopsis thaliana*) (Yao et al., 2022; Liu et al., 2023). Exogenously overexpressing *FvMYB82* from strawberry (*Fragaria vesca*) and the R1-MYB transcription factor gene *LcMYB1* from sheepgrass (*Leymus chinensis* (Trin.) Tzvel.) enhanced the salt tolerance of transgenic Arabidopsis (Cheng et al., 2013; Li et al., 2022a). *AgMYB5*, an unknown gene from celery (*Apium graveolens L.*), enhanced β-carotene synthesis in transgenic Arabidopsis (Sun et al., 2023)(**Figure 1A**). Among NAC family transcription factor genes, overexpressing *CaNAC46* from pepper (*Capsicum annuum*) and *SlNAC10* from *Suaeda liaotungensis* enhanced the salt and drought resistance of transgenic Arabidopsis (Ma et al., 2021; Du et al., 2022) (**Figure 1B**). Among genes in the WRKY transcription factor family, *OsWRKY54* is associated with salt tolerance in rice; heterologous expression of *VvWRKY28* from grapevine (*Vitis vinifera*) and *PcWRKY11* from *Polygonum cuspidatum* in Arabidopsis enhanced salt tolerance (Liu et al., 2022b; Wang et al., 2022a) (**Figure 1C**).

The other type is genes without a domain and with unknown functions. Several unknown genes that lack typical conserved domains also function in plant growth, development, and resistance to stress. The U/U gene *Lb1G04202* from the halophyte *Limonium bicolor*, which lacks known structural domains or special structures, encodes a protein that functions in the nucleus and enhanced NaCl tolerance in Arabidopsis by alleviating osmotic stress. The RNA of *Lb1G04202* localizes to the salt gland (a unique salt-secreting structure) of *Limonium bicolor*, suggesting that this gene plays a role in salt gland development (Wang et al., 2022b). In a word, U/U genes with and without conserved domains play significant roles in plants.

## Methods to study U/U genes

U/U genes are almost always discovered in non-model plants, making functional studies quite challenging due to unstable transformation systems. Whole genome sequencing and comparative genomics are essential techniques for performing functional studies of these genes (Geng et al., 2022; Liu and Zhang, 2022; Yuan et al., 2022). U/U genes are always identified by RNA-seq, but their assembled sequences are not always accurate. Therefore, transcriptome sequencing techniques such as Iso-seq that yield full-length reference sequences are recommended in studies examining U/U function (Yuan et al., 2015; Yuan et al., 2016; Jia et al., 2022). Iso-seq can directly obtain complete transcripts without disrupting splicing, in order to accurately analyze structural information such as variable splicing and fusion genes of reference genome species. This technology can also promote the optimization of genome annotation and quantification of transcriptome abundance, providing opportunities for the discovery of new genes (Rhoads and Au, 2015; Li et al., 2017; Beiki et al., 2019; Jia et al., 2022).

Map-based cloning can be used to isolate and clone plant genes and to localize genes on chromosomes (Lee et al., 2019). This technology is particularly suitable for situations where the expression products of genes are unknown, functional information for unknown genes is lacking, or no suitable phenotypes are observed (Jin et al., 2022; Zhan et al., 2023). However, the complete sequence of new genes cannot be fully mastered, which undoubtedly poses difficulties for the full-length cloning and isolation. RACE (rapid amplification of cDNA ends) is an effective method for studying new genes, which based on PCR and RNA reverse transcription. It rapidly amplifies the unknown sequence regions of the 3’ or 5’ ends of cDNA through partial known gene sequences to obtain full-length cDNA (Groot Kormelink and Luyten, 1997; Lindberg et al., 1997; Cheng et al., 2006; Yeku and Frohman, 2011).

Bioinformatics analysis of candidate genes is crucial, as it provides a rough understanding of the possible range of gene action through domain prediction (SMART), hydrophilicity analysis (Expasy-ProtScale) (Dong et al., 2022), transmembrane region display (TMHMM 2.0) (Zhao et al., 2022), and subcellular localization prediction (WoLF PSORT) (Wang et al., 2021; Song et al., 2022). Bioinformatics analysis can lay a solid foundation for further in-depth research of U/U genes (Chen et al., 2022).

RNA interference (RNAi) is an efficient tool for studying the effects of gene deletions (Koeppe et al., 2023; Traber and Yu, 2023). Gene silencing mediated by double-stranded RNA (dsRNA) is widely used to study gene functions in various plants (Akond et al., 2022; Bharathi et al., 2023). Another efficient method to identify gene function is clustered regularly interspaced short palindromic repeats (CRISPR)/CRISPR-associated nuclease 9 (Cas9)-mediated gene knockout (Hu et al., 2023). The phenotypes obtained using these two methods can be compared to phenotypes obtained via overexpression to analyze the biological function of the target gene or protein (Yuan et al., 2022). Of course, it is not sufficient to conduct research solely in the species harboring U/U genes. The transfer of candidate genes into model plants (*Arabidopsis*) or prokaryotic bacteria is extensively used for further functional research (Leng et al., 2021; Wang et al., 2022b).

The completion of various life functions in plant cells relies on interactions between proteins (Beihammer et al., 2023). Typically, functional proteins combine with other proteins (known or unknown) to form complexes and function in specific pathways (Zhao et al., 2008). Therefore, it is necessary to identify genes that are involved upstream or downstream of the U/U gene of interest and validate the interactions between their encoded proteins. U/U proteins and candidate proteins that may interact with each other identified by screening yeast libraries can be validated by examining *in vitro* and *in vivo* interactions using yeast-two hybrid assays (Cao et al., 2022), bimolecular fluorescence complementation (BiFC) (Choi et al., 2022), co-Immunoprecipitation (CoIP), and GST-pulldown (Du et al., 2023) in order to elucidate the associated signaling pathways (Liu et al., 2022a).

## The roles of U/U genes in regulating plant growth

**Figure 2:**
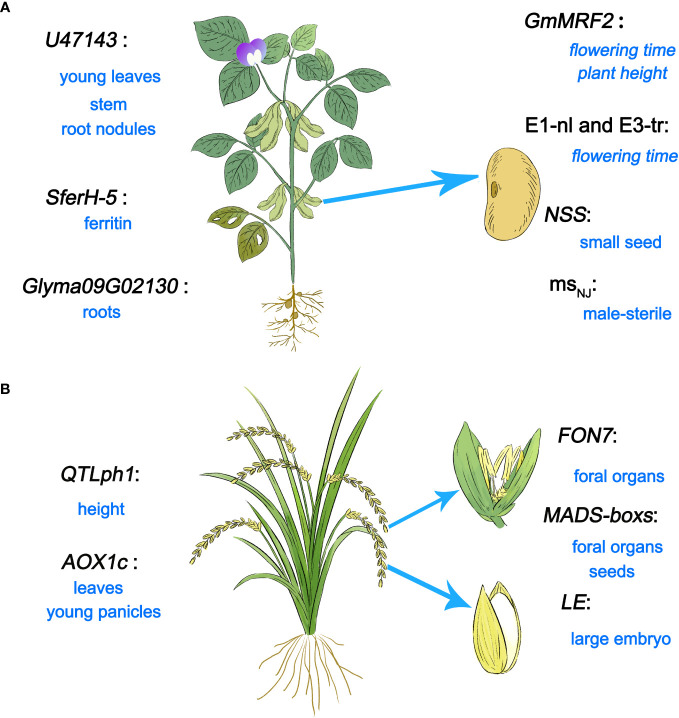
U/U genes related to plant growth regulation. **(A)** U/U genes related to growth and development identified in soybean. **(B)** Newly identified genes in rice and their functions in growth and development.

U/U genes that regulate plant growth and development are distributed across a variety of species, particularly soybean (*Glycine max*), rice (*Oryza sativa*), and non-model plants. The functions of reported U/U genes throughout the lifecycles of soybean and rice are shown in **Figure 2**.

### U/U genes in soybean

To date, many U/U genes that participate in reproductive growth have been identified in soybean (**Figure 2A**). The hemoglobin gene *U47143* from soybean shares a protein sequence similarity of only 58% with another hemoglobin gene in soybean. As soybean is a non-symbiotic leguminous plant, hemoglobin is expressed in various tissues of this plant, such as cotyledons, seedling stems, roots, young leaves, and root nodules (Anderson et al., 1996). *Glyma09G02130*, a new NHX (Na^+^/H^+^ antiporter gene family) gene, was identified from the soybean genome, which is related to root growth. Under salt stress, the expression level of *Glyma09G02130* in roots is significantly upregulated (Chen et al., 2015). A new ferritin gene *SferH-5* has been cloned from soybean seedlings, which is related to the production of ferritin in soybean (Dong et al., 2007). The MORN motif type gene *GmMRF2* has also been isolated from soybean. Soybean lines overexpressing *GmMRF2* exhibited earlier flowering under long day (LD) conditions and showed an increase in plant height under both LD and short day (SD) conditions. In addition, gibberellin pathway genes which positively regulate plant height and promote flowering, were significantly upregulated in *GmMRF2*-overexpression lines (Zhang et al., 2023a), reflecting the important roles of *GmMRF2* in regulating flowering time and plant height. In addition, *E1-nl* and *E3-tr* were identified as related to flowering time by AmpliSeq technology (Ogiso-Tanaka et al., 2019). By analyzing T-DNA mutant (*S006*) seeds, a gene related to seed development was discovered, named *New Seed Size* (*NSS*). Seeds of the CRISPR/Cas9-generated *nss1* mutant were small with brown seed coats, which is consistent with the phenotypes of *S006* seeds (Zhang et al., 2023b). What’s more, a novel male-stelile gene *ms_NJ_* has been discovered (Nie et al., 2019). In a word, U/U genes are distributed at various locations in soybean and participate in different life activities.

### U/U genes in rice

A gene underlying a quantitative trait locus (QTL) controlling plant height on chromosome 1 (*QTLph1*) was identified in rice; this gene encodes a protein that promotes sucrose transport to the leaves (Ishimaru et al., 2004) (**Figure 2B**). Ten new MADS-box homologous genes were identified in rice using pan-genome, all of which were expressed in flower tissue and six were highly expressed during seed development (Li et al., 2023a). A novel gene encoding alternating oxidase (*AOX1c*) was isolated from rice, mainly expressed in leaves and young panicles (Saika et al., 2002). The U/U gene Os08g0299000, named *FLORAL ORGAN NUMBER7 (FON7)*, was identified in a mutant with altered floral organ number (generated by ethyl methanesulfonate treatment of Korean *japonica* rice cultivar Ilpum); this gene controls the number of floral organs. The *fon7* mutant shows an increased number of stamens and pistils. The number of floral organs plays crucial roles in fruit development and grain maturity (Maung et al., 2023). In rice, *LARGE EMBRYO (LE)*, a U/U gene that controls embryo size, was identified and characterized. In *le* mutants and RNA interference lines, the embryo size is increased, indicating that *LE* plays a decisive role in controlling embryo size (Lee et al., 2019). Therefore, U/U genes in rice play a major role in growth and reproduction.

### U/U genes in other plants

Most other U/U genes have been reported in Arabidopsis, poplar, and sweet potato (*Ipomoea batatas* Lam). Four *Arabidopsis* mutants (*rem1.2*, *orc1a*, *ppd1*, and *mcm4*), exhibit varying degrees of reduction rosette size, confirming the novel role of these U/U genes in effective leaf surface area (ELSA) (Gonzalez et al., 2020). In Arabidopsis, the line expressing COBRA gene family showed a significant decrease in cellulose content, and the new member was identified related to the secondary cell wall formation (Brown et al., 2005). The protein encoded by the U/U transcription factor gene *PebHLH35* (from *Populus euphratica*) enhances drought resistance by regulating stomatal development and photosynthesis, as demonstrated in transgenic Arabidopsi*s* plants heterologously expressing this gene (Dong et al., 2014). The protein encoded by the U/U sucrose transporter gene *IbSUT4* from sweet potato participates in plant growth by intervening in the abscisic acid signaling pathway (Wang et al., 2020). The U/U *BrSCC1* gene *BraA03g040800.3C* identified in *Brassica rapa* L has been verified to be related to the seed coat color (Zhang et al., 2023c). The U/U gene *GhMPK7* has been isolated from cotton, whose overexpression in transgenic tobacco promoted the transcription level of SA pathway quickly and efficiently and showed earlier germination compared to WT (Shi et al., 2010). It can be seen that there are numerous U/U genes distributed in different plants and participated in various life activities.

## The roles of U/U genes in abiotic stress resistance

**Figure 3:**
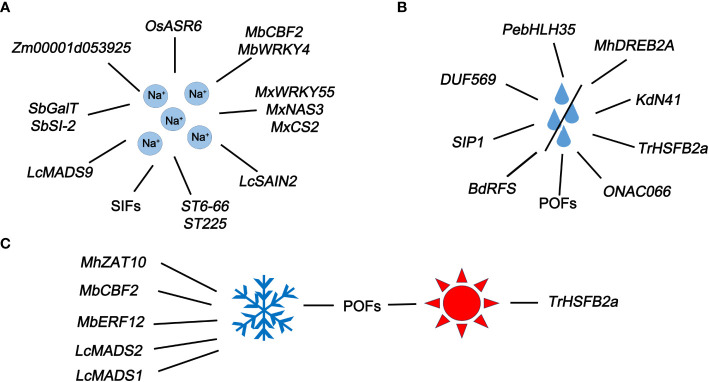
U/U genes closely related to abiotic stress. **(A)** U/U genes that respond to Na^+^ in plants. **(B)** U/U genes that respond to water deficiency in plants. **(C)** U/U genes that respond to extreme temperatures.

Most U/U genes identified in different plant play function in responses to different types and degrees of stress, including high salt (**Figure 3A**), water scarcity (**Figure 3B**), and harsh temperatures (high or low) (**Figure 3C**).

### Salt stress

In rice, *OsASR6* (a newly identified salt-induced ASR gene) is upregulated under salt stress. *OsASR6* RNAi transgenic lines showed poorer salt tolerance and oxidative stress capacity than the untransformed control plants, while genetically modified rice lines with *OsASR6* overexpression showed excellent performance (Zhang et al., 2022a). A U/U SIF gene in rice might be involved in the plant response to salinity stress (Soda et al., 2013). A new WRKY gene named *MxWRKY55* was isolated from *Malus xiaojinensis* and introduced into Arabidopsis to significantly improve its salt tolerance and biomass (Han et al., 2020). What’s more, overexpression of *MxNAS3* cloned from *M. xiaojinensis* in transgenic Arabidopsis improve biomass and root length. Importantly, the high expression of *MxNAS3* in transgenic Arabidopsis is associated with the formation of malformed flowers (Han et al., 2018b). Also, a new gene *MxCS2* encoding citrate synthase promotes the synthesis of citrate synthase and increases the content of CA in Arabidopsis. Overexpression of *MxCS2* also increased the fresh weight, root length, CS activity, as well as chlorophyll and citric acid content (Han et al., 2014)*. MbCBF2*, an uncharacterized gene from *Malus baccata* (L.) Borkh, increased its expression in young tissues under high salt induction. In heterologous overexpressed Arabidopsis lines, it can enhance the adaptation to high salt environment and change physiological indicators related to stress, including proline, malondialdehyde (MDA) and superoxide dismutase (SOD), which reflects the tolerance of *MbCBF2* to salt stress (Li et al., 2022b). Overexpression of a novel WRKY gene *MbWRKY4* in transgenic tobacco enhances salt tolerance (Han et al., 2018a).

Functional analysis of the salt cress (*Thellungiella halophila*) genes *ST6-66* and *ST225* in Arabidopsis revealed their importance in salt resistance (Du et al., 2008). The U/U gene *LcMADS9* was significantly upregulated in sheepgrass (*Leymus chinensis (Trin.)* Tzvel) under NaCl treatment, highlighting the response of this gene to NaCl (Jia et al., 2018). *LcSAIN2*, another salt-induced U/U gene from sheepgrass, enhanced salt tolerance in transgenic Arabidopsis plants (Li et al., 2013). Transcriptome sequencing of two maize (*Zea mays*) inbred lines revealed the U/U gene Zm00001d053925, whose expression level was significantly higher in AS5 (salt tolerant line) than in NX420 (salt intolerant line), indicating that Zm00001d053925 functions in the plant response to salt stress (Zhu et al., 2023) (**Figure 3A**). A U/U gene *galactosyl transferase*-like (*SbGalT*) from *Salicornia brachiata* alleviates osmotic and salt stress in transgenic tobacco (Dubey et al., 2021). Also, another new salt induced gene *SbSI-2* (*Salicornia brachiata salt-inducible-2*) has been functionally identified to have the same function as *SbGalT* (Pandey et al., 2014). In one word, U/U genes exercise significant functions in response to salt stress.

### Drought stress

In *Populus euphratica*, the transcription factor *PebHLH35* confers drought resistance by regulating various developmental and physiological processes (Dong et al., 2014). *DEHYDRATION RESPONSE ELEMENT-BINDING PROTEIN 2A* (*DREB2A*) in apple (*Malus domestica*) responds to drought stress and plants overexpressing *MhDREB2A* exhibited enhanced tolerance to drought (Li et al., 2023b). An uncharacterized *KdNOVEL41 (KdN41)* gene from *Kalanchoe (K.) daigremontiana* confers drought resistance on *K. daigremontiana* and tobacco (*Nicotiana tabacum*) by playing a role in clearing reactive oxygen species and reducing osmotic damage (Wang et al., 2018). The unique proteins with obscure features (POFs) of Arabidopsis enhance tolerance to oxidative stress, including osmotic, salinity, and temperature stress (Luhua et al., 2008). The U/U gene *BdRFS* of *Brachypodium distachyon* has been identified to be functionally conserved, together with improve the drought resistance of *Brachypodium* and *Arabidopsis* (Ying et al., 2023). The inactivation of *SIP1*, encoding an unknown protein in Arabidopsi*s*, decreased drought tolerance (Anderson and Kohorn, 2001). Furthermore, a novel gene *DUF569* (*AT1G69890*) with “domain of unknown function” positively regulates drought stress in Arabidopsis (Nabi et al., 2021). The HSF transcription factor gene *TrHSFB2a* (B-type HSF), which was recently identified in drought-sensitive white clover (*Trifolium repens*), negatively regulates drought resistance (Iqbal et al., 2022) (**Figure 3B**). Under drought stress conditions, overexpression of the *ONAC066* gene (a novel gene whose function has been newly determined) enhances the tolerance of rice to drought stress and sensitivity to ABA (Yuan et al., 2019). Numerous U/U genes responding to drought stress undoubtedly bring new possibilities for improving plant drought resistance.

### Extreme temperature stress

*MbCBF2*, a U/U CBF transcription factor gene from *Malus baccata* (L.) Borkh, shows elevated expression at low temperatures. Exogenously overexpressing *MbCBF2* enhanced the adaptability of transgenic Arabidopsis to cold conditions (Li et al., 2022b). *MbERF12*, an ERF gene, enhances its ability to scavenge reactive oxygen species through ethylene signaling, playing a crucial role in the response of salt and low temperature stress (Han et al., 2021). *ZINC FINGER OF ARABIDOPSIS THALIANA 10* (*ZAT10*), a U/U gene in *Malus domestica*, is activated under low temperature stress. Apple lines overexpressing *MhZAT10* showed increased tolerance to low temperature stress, indicating that this gene plays an important role in cold resistance (Li et al., 2023b). Low temperature significantly induced *LcMADS1* and *LcMADS2* expression in sheepgrass (Jia et al., 2018).

*TrHSFB2a* expression in white clover was strongly induced by exposure to high temperature (35°C) and the encoded protein negatively regulates heat tolerance (Iqbal et al., 2022). The POFs in Arabidopsis enhance plant tolerance to oxidative stress under both cold and heat stress (Luhua et al., 2008) (**Figure 3C**). Under harsh temperatures, in addition to previously characterized genes, there are also these uncharacterized genes, which enriches the large category of genes that have resistance to extreme temperature.

Though different new genes were identified in various stress, plants usually experience fluctuations in several key hormone levels during their early stress response, such as ABA, SA and JA (Verma et al., 2016). ABA is a regulatory factor for many plants under environmental stress, including drought, low temperature, and salinity. Abiotic stress generates osmotic signals, leading to ABA accumulation (Danquah et al., 2014). SnRKs are involved in osmotic stress and ABA signal transduction, and both SnRKs and ABA pathways involve MAPK responses (Zhu, 2016). Under extreme stress conditions, ROS is overproduced and causes oxidative damage to plants (Verma et al., 2016). After ROS signal transduction, anthocyanins are produced, which are used for antioxidant activities by clearing excess ROS (Naing and Kim, 2021). In short, plants have a similar fate after being subjected to abiotic stresses, which can trigger a series of homologous stress tolerance activities.

## The roles of U/U genes in biological stress responses

Plant diseases such as powdery mildew, bacterial blight, and leaf rust frequently occur in plants (especially food crops), which greatly reduces crop quality and yields. Many U/U genes in crops are related to diseases responses. Here we summarize progress in identifying genes involved in biological stress resistance. Among them, most do not have obvious domains, except for family genes such as NAC.

### Leaf rust resistance

Wheat leaf rust, a disease caused by *Puccinia triticina*, mainly damages the leaves of common wheat (*Triticum aestivum*) and causes serious losses in wheat production (Qi et al., 2023). Currently, the most effective control measure involves breeding and using resistant wheat varieties.

**Figure 4:**
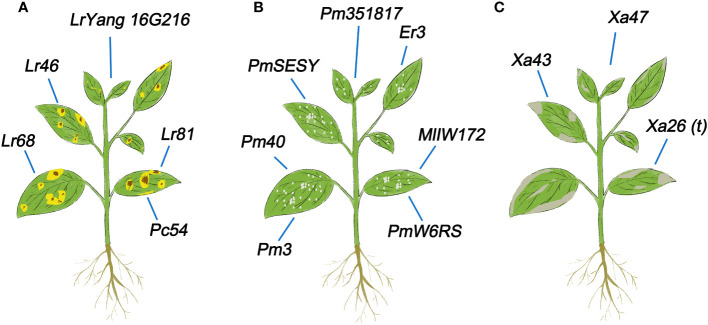
U/U genes related to resistance to biological stress in plants. **(A)** U/U genes involved in leaf rust resistance in plants. **(B)** U/U genes involved in powdery mildew resistance in plants. **(C)** U/U genes involved in bacterial blight resistance in plants.

The U/U gene *Lr68* in common wheat confers slow-rusting resistance to wheat rust, as demonstrated in the field (Herrera-Foessel et al., 2012). *Lr46* is also associated with slow-rusting resistance to leaf rust in wheat (Singh et al., 1998). A leaf rust resistance gene named *Lr81* was identified in wheat line PI470121, which is a unique leaf rust resistance locus (Xu et al., 2022a). A stable APR gene, named *LrYang16G216*, was detected in wheat and identified as a new and effective gene for leaf rust resistance (Zhao et al., 2023b). A gene *Pc54* with leaf rust resistance has been identified in oat (*Avena sativa*) (Admassu-Yimer et al., 2022).These newly identified genes all have excellent activity in inhibiting rust (**Figure 4A**), which could contribute to the breeding of rust resistant wheat varieties.

### Powdery mildew resistance

Powdery mildew is a crop disease induced by *Blumeria graminis* f. sp. Tritici that is extremely destructive to common wheat (Mapuranga et al., 2022). Identifying powdery mildew resistance genes could suggest strategies to improve growth and yield in wheat and other crops.

The U/U powdery mildew resistance gene *Pm40* of *Elytrigia intermedium* confers resistance to this disease and has been transferred to wheat to enhance its resistance to powdery mildew (Luo et al., 2009). A gene *Pm3* with powdery mildew resistance was identified in oat (*Avena sativa*) (Admassu-Yimer et al., 2022). *PmW6RS* has been identified as a powdery mildew resistant gene in rye (*Secale cereale L., RR*), providing a new gene selection for wheat disease resistance breeding (Wang et al., 2023). *Pm351817*, a new Pm65 allele in wheat, exhibits resistance to powdery mildew (Xu et al., 2023). *PmSESY* in rye (*Secale sylvestre*) also confers resistance to powdery mildew and can significantly improve resistance to this disease (He et al., 2021). The U/U gene *Er3*, which was identified in *Pisum fulvum*, markedly improves the resistance to powdery mildew (Sara et al., 2007). The U/U allele *MlIW172* of *Pm60* was shown to enhance resistance to powdery mildew in wheat by transgenic complementation (Wu et al., 2022). These genes provide genetic diversity for breeding wheat with enhanced resistance to powdery mildew (**Figure 4B**).

### Bacterial blight resistance

Bacterial blight (BB), a disease caused by *Xanthomonas oryzae* pv. oryzae (Xoo), is a serious rice disease worldwide (Javed et al., 2022). Therefore, identifying and isolating BB resistance genes from different rice resources is of great significance. Different rice varieties have multiple different BB resistance genes. The BB resistance gene *Xa43* was recently identified in Zhangpu wild rice (*Oryza rufipogon*) (Huang et al., 2023). A new NLR disease resistance gene *Xa47* has long-term resistance to rice BB disease (Lu et al., 2022b). *Xa26(t)*, which was identified in rice variety Minghui 63, has a dominant effect on the Chinese Xoo strain JL691 at both the seedling and adult stages (Yang et al., 2003) (**Figure 4C**). It can be seen that the identification and utilization of U/U genes are of great significance for resisting BB.

### Resistance to other diseases

Multiple resistance genes to downy mildew exist in wild *Lactuca*, 11 of which were introduced into lettuce (*Lactuca sativa L.*) to facilitate the development of multi-gene downy mildew resistant lines (Parra et al., 2020). The resistance gene *Rsg3* was recently discovered in Chinese barley landrace PI 565676 (a landrace from China). This gene, which provides strong resistance to greenbug (*Schizaphis graminum Rondani*), should help alleviate the major threat of this insect pest to global food production (Xu et al., 2022b). The resistance gene *bph42*, which confers resistance to brown planthopper (BPH), was identified in wild rice line *Oryza rufipogon* (*Griff.*) and transferred to cultivated rice (*Oryza sativa*), laying the foundation for the production of high-quality rice with enhanced insect resistance (Kaur et al., 2022). *Brassica rapa* shows obvious resistance to turnip mosaic virus (TuMV). Through genetic analysis, a uncharacterized TuMV resistance gene, *BraA06g035130.3C*, was recently identified, paving the way for improving TuMV resistance and agricultural production (Lu et al., 2022a). The U/U gene *GbNAC1* from *Gossypium barbadense L.* has been identified to be positively involved in the regulation of *Verticillium Wilt* resistance (Wang et al., 2016).

## Perspectives

More than a quarter of genes in the genomes of both crops and halophytes encode proteins of unknown function (Luhua et al., 2008). Some of these genes encode at least one previously defined domain or motif, but most lack previously defined features. Although transcriptome, metabolome, and proteome data show that many of these genes play important roles in plant growth, metabolism, physiology, and other life processes, their functions remain to be identified.

Nowadays the model organism Arabidopsis can be used to verify the functions of these genes via heterologous transformation and other experimental techniques, but studies of unknown genes should focus on their functions in the species harboring these genes and establishing genetic transformation systems for these species. Generating overexpression and silencing lines of the target gene of the species of interest via genetic transformation and observing the phenotypes of the transgenic lines would enable the analysis of gene function more directly and accurately. The functional study of unknown genes is not limited to the genes themselves. Genes are usually regulated by key upstream factors, and they encode proteins that regulate downstream genes. Therefore, clarifying the functions of the upstream and downstream factors of U/U genes and establishing a complete gene regulatory network are important aspects of functional studies of these genes.

U/U genes not only encode proteins that perform various biological functions in plants, but they also play important roles in the life activities of animals, microorganisms, and especially humans. We can also find inspiration from the study of U/U genes in animals. FREPs, a recently identified gene family in mussels (*Mytilus edulis*), are related to immune recognition in mollusks (Gorbushin and Iakovleva, 2011). A recently identified Ig kappa gene in sea star (*Asterias rubens*) confers specific resistance to horseradish peroxidase (Vincent et al., 2014). Previously unidentified genes obtained from chromosome replication promoted the study of the Neuropeptide Y family in vertebrates (Sundström et al., 2008). *Innexin 3*, a gene involved in dorsal closure in embryos, has also been identified in *Drosophila* (Fanning et al., 2013). A newly discovered gene that confers resistance to influenza virus H5N1 was identified in duck (*Anas platyrhynchos*) through transcriptome analysis (Huang et al., 2019). A new human membrane-associated mucin of the ocular surface was recently identified, which could contribute to the protection of human eyes (Fini et al., 2020). The discovery of a series of new genes in males revealed a new pathway for the production of testosterone (Flück and Pandey, 2014). New genes that function in osmotic stress resistance in the yeast *Saccharomyces cerevisiae* have also been identified (Gonzalez et al., 2016).

The study of U/U genes faces challenges because it is often unclear to which pathways these genes contribute. We can also uncover the unexpected functions of U/U genes in plant development and resistance, providing essential information to supplement our knowledge of known functional genes and improve our understanding of the connections between biological molecules.

In summary, numerous uncharacterized genes in living organisms have yet to be discovered, isolated, analyzed, cloned, and functionally identified. Some of these genes play key roles in the lifecycles of living organisms. Exploring these genes may enrich our understanding of existing physiological processes, metabolic pathways, and functional networks and offer new strategies to modulate them.

## Author contributions

XW: Writing – original draft. BW: Writing – review & editing. FY: Writing – review & editing.
